# The Effectiveness and Safety of Medical Cannabis for Treating Cancer Related Symptoms in Oncology Patients

**DOI:** 10.3389/fpain.2022.861037

**Published:** 2022-05-20

**Authors:** Joshua Aviram, Gil M. Lewitus, Yelena Vysotski, Mahmoud Abu Amna, Anton Ouryvaev, Shiri Procaccia, Idan Cohen, Anca Leibovici, Luiza Akria, Dimitry Goncharov, Neomi Mativ, Avia Kauffman, Ayelet Shai, Gil Bar-Sela, David Meiri

**Affiliations:** ^1^Faculty of Biology, Biology Department, Technion-Israel Institute of Technology, Haifa, Israel; ^2^Cancer Center, HaEmek Medical Center, Afula, Israel; ^3^Department of Oncology, Galilee Medical Center, Nahariya, Israel; ^4^Azrielly Faculty of Medicine, Bar Ilan University, Zafed, Israel; ^5^Faculty of Medicine, Technion-Israel Institute of Technology, Haifa, Israel

**Keywords:** medical use, cannabis, phytocannabinoids, oncology, cancer, prospective

## Abstract

The use of medical cannabis (MC) to treat cancer-related symptoms is rising. However, there is a lack of long-term trials to assess the benefits and safety of MC treatment in this population. In this work, we followed up prospectively and longitudinally on the effectiveness and safety of MC treatment. Oncology patients reported on multiple symptoms before and after MC treatment initiation at one-, three-, and 6-month follow-ups. Oncologists reported on the patients' disease characteristics. Intention-to-treat models were used to assess changes in outcomes from baseline. MC treatment was initiated by 324 patients and 212, 158 and 126 reported at follow-ups. Most outcome measures improved significantly during MC treatment for most patients (*p* < 0.005). Specifically, at 6 months, total cancer symptoms burden declined from baseline by a median of 18%, from 122 (82–157) at baseline to 89 (45–138) at endpoint (−18.98; 95%CI= −26.95 to −11.00; *p* < 0.001). Reported adverse effects were common but mostly non-serious and remained stable during MC treatment. The results of this study suggest that MC treatment is generally safe for oncology patients and can potentially reduce the burden of associated symptoms with no serious MC-related adverse effects.

## Introduction

Many comorbidities are associated with oncology diseases. Cancer-associated symptoms include pain (1), anxiety (2), depression (3), insomnia (4), decreased quality of life (5), increased disability (6) and negative effects on sexuality (7). These symptoms are some of the most fundamental causes of oncology patients suffering and disability while undergoing therapies, and some may even lead to worsened prognosis (3). Though well-known and documented, there is no optimal treatment for all of these comorbidities as of yet (8). Traditionally, cancer-related pain is mainly treated by opioid analgesics. In a recent Cochrane collaboration review of opioids for cancer pain, which thoroughly assessed 152 studies with 13,524 patients, treatment success was reported by 95% of patients, but most did not assess pain reduction adequately. Moreover, Wiffen et al. (9) concluded that the quality of evidence in favor of opioid treatment is poor, as the studies on which the treatment decision was based were old and with small sample size, and adverse events rates ranged from 11 to 77% (9). That is probably one of the reasons why most oncologists perceive opioid treatment as hazardous and alternative therapies are required.

A promising substitute for opioid-based medication is medical cannabis (MC). However, there is a knowledge gap in the study of cannabis, especially for treating cancer-related pain, and results are controversial. Thus far, only a few randomized controlled trials (10–16) and even fewer cohorts (17–19) investigated the effects of cannabinoids on cancer-related pain and scantly also on other cancer symptoms. Consequently, these findings led to a weak recommendation for utilizing cannabinoids for cancer pain treatment (20). However, although these studies were randomized controlled trials, most of them consisted of a small sample size and additional studies are needed (21). A more recent meta-analysis showed no favorable effect for Nabiximols in cancer pain (22). Nevertheless, a recent study showed that most cancer patients requested MC treatment from their oncologist (23).

The adverse effects (AEs) from cannabinoids for cancer treatment are generally well tolerated by the patients and categorized as mild to moderate. The most frequent AEs are memory impairment, drowsiness, nausea, vomiting and xerostomia (dry mouth). Cannabinoid treatment for cancer-related pain is generally recognized as safe (24). Nonetheless, drug-drug interactions should be taken into account. Recent retrospective and prospective studies showed decreased response rates when immunotherapy was administered concomitantly with an MC treatment (25, 26). Although a previous prospective analysis was conducted on cancer patients following 6–8 weeks of treatment (17), it lacked the added value of repeated observation by multiple follow-up points, the utilization of validated questionnaires, specific MC treatment characterization and longer follow-up. Thus, we conducted a multi-center, prospective, 6-month longitudinal study that followed up on the effectiveness and safety parameters of MC treatment for cancer-associated symptoms. The approach of real-world evidence undertaken in the study provides prospective and structured data collection, and allows the data mining of many patients from real-world data, as is especially important for cancer patients that commonly suffer from associated comorbidities. Similar approaches have proven very useful in assessing the effectiveness of medical treatments in the fields of trauma, cancer, cardiology, stroke and arthritis (27). Similarly, worldwide opioid registries have assisted in reducing treatment-related mortality (28) and in assessing the treatment's long-term effectiveness (29).

## Methods

### Israeli Medical Cannabis Regulations

The Israel Ministry of Health (IMOH) regulations allow issuing an MC license to treat palliative phase cancer patients and cancer patients with antineoplastic treatment-related adverse effects (30). Licenses for MC use are issued by specific oncologists that received a personalized mandate from the IMOH. The issuing oncologist then prescribes the MC dose (grams per month), route of administration and the cannabidiol (CBD) and (-)-Δ^9^-*trans*-tetrahydrocannabinol (THC) concentrations (30), based on the IMOH guidelines. Two routes of MC consumption are approved: inflorescences (for smoking or inhaling) and/or oil extracts (for sublingual use). The initial dose is 20 gr/month regardless of the route of administration. At the time this study was conducted, MC was purchased at pharmacies and was non-reimbursable. The official contraindications for MC in Israel were pregnancy, lactation, and previous psychotic diagnosis or family history of psychotic illness.

### Study Design

This multi-center, prospective, long-term study was conducted between January 2019 and September 2021. The institutional Ethics Committee of Haemek Medical Center (#0137-18-EMC) and Galil Medical Center (#0010-19-NHR) approved the study. This was a pure observational study with no interventional component whatsoever, so registration at the Clinical Trials Register was not required. Importantly, no recognizable information on participating patients is published anywhere in this research paper.

Hebrew-speaking patients aged >18 years licensed for MC for treating any form of cancer-related symptoms for the first time were eligible to participate in the study. After explaining the study procedures, oncologists who agreed to participate (all are co-authors in the current study) and regularly issue MC licenses, obtained written informed consents from eligible patients. Copies of the consent forms along with the patients' contact information were sent to the study coordination center. To avoid any possible influence of the collected data on the physicians' decisions regarding the clinical management of their patients, prescribing physicians had no access to data collected on individual patients.

Patients were instructed to complete the study questionnaires at baseline, before MC treatment initiation (T_0_; up to a few days before), and at three follow-up times: one (T_1_), three (T_3_), and six (T_6_) months following treatment initiation. The questionnaire consisted of 174 questions at baseline and a variable number of about 220 at each follow-up. Questions were presented in a dynamic format customized to individual responses, where responses to a particular question determined the subsequent questions asked. To reduce the study burden, patients were given a choice to skip questions. Hence, each patient completed a unique set of questions and each question received a different number of responses. Data was collected online by the secured survey technology Qualtrics® (Provo, Utah, version 12018) (31). Whenever patients had difficulties using the web platform, the questionnaires could be completed by phone with the assistance of the study coordinator. No financial compensation was offered to participating patients. The STROBE statement checklist for cohort studies is presented in the Supplementary Table 1.

### Study Questionnaires

Oncologist reported information included cancer diagnosis, classification of malignant tumors (TNM), cancer treatment protocol and the Eastern Cooperative Oncology Group (ECOG) Performance Status score (32). Patient-reported information included demographics, analgesics consumption, MC treatment characteristics as well as Hebrew validated oncology-related questionnaires, including (1) the study's primary outcome measure of the total sum of Memorial Symptom Assessment Scale (MSAS) of cancer symptoms burden (33); (2) average weekly pain intensity on a 0–10 numerical pain scale (NPS) and the weekly average worst and least pain intensities; (3) The short-form McGill Pain Questionnaire (SF-MPQ) (34); (4) Quality of life - EuroQol (EQ5) (35); (5) Pittsburgh Sleep Quality Index (PSQI) (36); (6) Beck Depression Inventory-II (BDI-II) (37); (7) Pain Catastrophizing Scale (PCS) (38); (8) General Anxiety Disorder (GAD-7) (39); and (9) the Female sexual function index (FSFI) (40), males received a modified version. Using a predetermined list (41), patients were requested to report on adverse effects they could attribute directly to MC use at each follow-up time-point. AEs were later classified as serious or non-serious, according to the FDA's definition (42).

### Phytocannabinoids Dose Assessment

Since the IMOH reform (43), MC cultivators in Israel are required to accurately indicate the THC and CBD concentrations in their products (30). We calculated the monthly doses of THC and CBD only for patients who reported fully on their MC treatment regimen, according to the products they reported consuming, based on their total and specific monthly doses. For example, if a patient reported consuming 10 g of THC10/CBD10 product and another 10 g of THC20/CBD4 product, the patient's calculated monthly consumption is 6,000 mg and 2,800 mg of THC and CBD, respectively. Notably, MC cultivators in Israel are not required to report on phytocannabinoids other than THC and CBD or terpenoids concentrations in their MC products.

### Statistical Analysis

R software (V.4.0.4) with lme4 (44), atable (45), and tidyverse (46) packages were used to analyze changes in outcome measures by linear mixed-effect regression models to assess the duration effect of the treatment (47). Due to the heterogeneity of the study characteristics, only intention to treat (ITT) analyses were conducted. Due to the prospective, longitudinal data collection design, each of the time points had a different sample size and was analyzed with the corresponding baseline information. In cases that had no differences in the measures between time points, the text relates to T_0_ data. Chi-square or Kruskal-Wallis rank tests were conducted to establish the similarity of demographic data between the three follow-ups. For the effect of size and confidence interval (CI), a Cohen's d test was utilized. The Shapiro-Wilk test of normality demonstrated non-normal distribution for all measures. Thus, data are presented as median (IQR, Q1–Q3, i.e., quartiles 25 and 75). Differences were considered significant at the *p* < 0.05 level. Incidences are presented as numbers and percentages of patients. The required minimum sample size was calculated for the study by G^*^Power statistical analysis (48), accounting for the following: four time-point repeated measures analysis, within factor analysis, medium effect size (0.25), α ≤ 0.05, power of 0.80 and 14 observables (all measured parameters). Based on these, a sample size of 56 patients was determined as appropriate. Notably, due to the exploratory nature of the study and many potential subgroup analyses, no maximum sample size objective was determined.

## Results

### Sample

**Figure 1:**
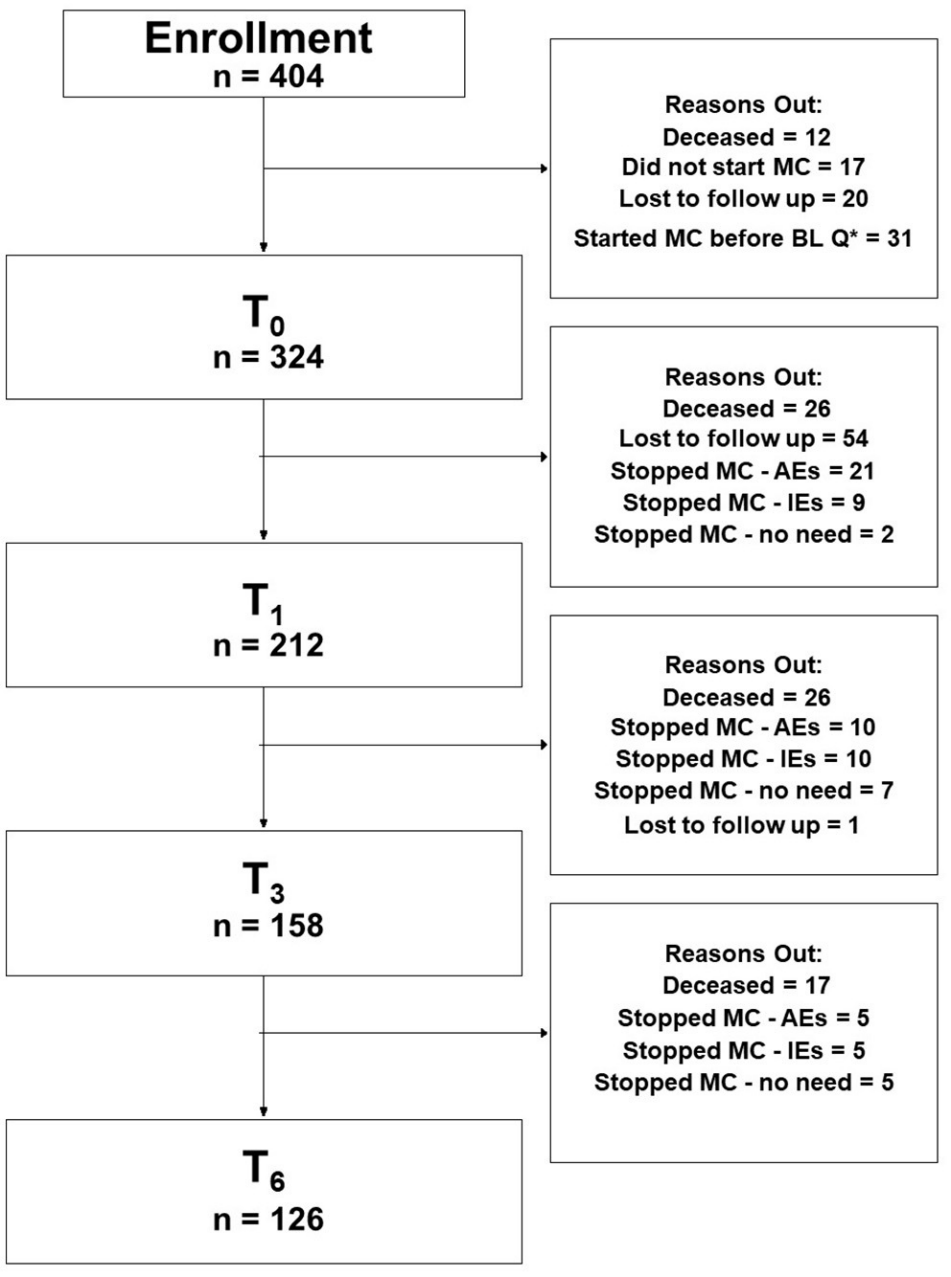
Consort flowchart diagram. MC, medical cannabis; Q*, questionnaire; BL, baseline; AEs, adverse effects; IEs, ineffectiveness; T_0_, baseline time point (prior to MC treatment initiation); T_1_, 1-month follow-up; T_3_, 3-month follow-up; T_6_, 6-month follow-up; PP analyses refer to patients that answered the study questionnaires at baseline and at all follow-up time points.

A total of 404 patients enrolled in the study following the acceptance of an MC license and obtaining pharmacy prescriptions. Of those, 80 (20%) were not eligible for further analyses due to the below-mentioned reasons (Figure 1, CONSORT flow diagram). Of the remaining 324 patients that initiated MC treatment and completed the baseline (T_0_) questionnaires, follow-up questionnaires were completed by 212 (at T_1_), 158 (T_3_), and 126 (T_6_) patients. The following reasons led to the decline in the number of participants over time: lost to follow-up [55 patients (17%)], ceased MC treatment due to ineffectiveness [24 patients (7%)], and due to MC-related AEs [36 patients (11%)], and due to no further need [e.g., chemotherapy-induced AEs stopped; 14 patients (4%)]. All of the patients that stopped MC treatment for the abovementioned reasons were alive at the 6-month follow-up period. Notably, 69 patients (21%) passed away during the follow-up period. About 98% of the patients provided data online and the rest by telephone calls.

### Sensitivity Analyses for Eligibility Criteria

Baseline demographic characteristics did not differ between eligible (*n* = 324) and non-eligible (*n* = 80) patients for age, gender, comorbidities, or overall analgesics consumption (Supplementary Table 2). Mentionable, eligible patients were more likely to have breast or colon cancer diagnoses than non-eligible patients.

### Baseline Demographics and Cancer Characteristics

Patients in the sample were on average 64 (49–68) years old and the majority (59% *n* = 192) were females. Previous exposure to cannabis was reported by 20% (*n* = 65) (Table 1). Oncology diagnoses were diverse, with breast cancer being the most frequent diagnosis (*n* = 89, 27%), followed by colon, lung and ovarian cancers (*n* = 32, 10%, *n* = 36, 11%, and *n* = 23, 7%; respectively). Most patients (*n* = 154, 48%) were categorized as stage IV cancer. Chemotherapy was the most prevalent current treatment protocol (*n* = 179, 55%). Most patients (*n* = 229, 71%) were scored by their oncologist as not disabled (scored ≤ 1 based on Eastern Cooperative Oncology Group (ECOG) Performance Status score) (Table 1). Most demographic measures did not change during the 6 months follow-up in the ITT cohort. Notably, the rate of patients with comorbidities decreased from baseline (χ^2^_(3)_ = 20.00, *p* < 0.001). More elaborated oncology diagnoses are presented in Supplementary Table 3.

**Table 1:** Demographic and cancer characteristics of the cohort at all time points.

**Parameters**	**T_**0**_ (*n =* 324)**	**T_**1**_ (*n =* 212)**	**T_**3**_ (*n =* 158)**	**T_**6**_ (*n =* 126)**	**(χ^2^)/ Kruskal-Wallis rank(*P* value)**
**Median (IQR)**					
**Age (years)**	64 (53–72)	64 (52–71)	62 (50–70)	61 (51–70)	2.97^††^ (0.39)
Missing N	26	13	11	9	
**Weight (kg)**	70 (61–81)	70 (59–80)	70 (60–80)	70 (62–80)	0.71^††^ (0.87)
Missing N	15	38	32	24	
**BMI**	25 (22–29)	25 (22–28)	26 (22–28)	25 (22–28)	0.62^††^ (0.89)
Missing N	24	51	40	32	
**No. of patients (%)**					
**Gender**					
Female	192 (59)	131 (62)	98 (62)	78 (62)	0.57^†^ (0.90)
Male	132 (41)	81 (38)	60 (38)	48 (38)	
Missing N	0	0	0	0	
**Comorbidities (yes)**	167 (52)	71 (33)	60 (38)	53 (42)	20.00^†^ (<0.001)
Missing N	11	8	3	1	
**Tobacco smoking at BL (yes)**	69 (21)	47 (22)	38 (24)	33 (26)	1.44^†^ (0.70)
Missing N	12	5	6	4	
**Previous cannabis experience (yes)**	65 (20)	48 (23)	39 (25)	33 (26)	1.98^†^ (0.58)
Missing N	9	0	0	0	
**Solid tumor etiology**					
Breast	89 (27)	63 (30)	54 (34)	42 (33)	5.40^†^ (0.94)
Colon	32 (10)	25 (12)	15 (10)	13 (10)	
Lung	36 (11)	24 (11)	12 (8)	11 (9)	
Ovaries	23 (7)	14 (7)	9 (6)	9 (7)	
Other	138 (43)	84 (40)	65 (41)	48 (38)	
Missing N	6	2	3	3	
**Solid tumor stage**					
I	19 (6)	16 (8)	15 (10)	13 (10)	6.63^†^ (0.68)
II	59 (18)	43 (20)	31 (20)	27 (21)	
III	53 (16)	35 (17)	27 (17)	21 (17)	
IV	154 (48)	97 (46)	63 (40)	47 (37)	
Missing N	39	21	22	18	
**Oncological treatment** ^**†**^					
Chemotherapy	179 (55)	117 (55)	85 (54)	73 (58)	0.25^†^ (0.97)
Biological	48 (15)	30 (14)	24 (15)	21 (17)	0.34^†^ (0.95)
Radiation	14 (4)	7 (4)	7 (4)	6 (5)	0.52^†^ (0.91)
Hormone	39 (12)	29 (14)	28 (18)	25 (20)	5.84^†^ (0.12)
Immunotherapy	24 (7)	17 (8)	12 (8)	11 (9)	0.23^†^ (0.97)
Missing N	2	4	4	0	
**ECOG score**					
≤ 1	229 (71)	157 (74)	122 (77)	98 (78)	3.36^†^ (0.34)
≥2	86 (27)	50 (24)	33 (21)	25 (20)	
Missing N	9	5	3	3	

*BMI, Body mass index; †, Pearson's Chi-squared test; ††, Kruskal-Wallis rank-sum test; IQR, Inter-quartile range; T_0_, baseline; T_1_, One-month Follow-Up; T_3_, Three-month Follow-Up; T_6_, Six-month Follow-Up; N, Number of patients; Yes, the number of subjects (%) who responded positively to the question; Percentages are rounded and without decimal points; kg, kilograms; ECOG, Eastern Cooperative Oncology Group Performance Status; †, numbers do not add up to 100% due to concomitant treatments*.

### MC Treatment Characteristics

Most MC treatment measures did not differ significantly during the six-month treatment. At the endpoint, MC oil extract was the most common route of administration (*n* = 52, 41%), consumed mostly sublingually. Although total monthly MC dose remained stable at a median (IQR) of 20 (20) gr, there was a significant increase that can be observed by the Mean ± SD from 21 ± 6.4 gr at T_1_ to 23 ± 6.3 gr at T_6_ (χ^2^_(2)_=8.55, *p* < 0.05). THC-rich cultivars were consumed more frequently, with monthly doses of THC increasing from 2,000 (1,000–3,500) mg at T_1_ to 3,000 (2,000–4,000) mg at endpoint (χ^2^_(2)_ = 3.12, *p* < 0.01). CBD monthly doses did not change significantly during the study (Table 2).

**Table 2:** Medical cannabis treatment characteristics.

**Parameters**	**T_**1**_ (*n =* 212)**	**T_**3**_ (*n =* 158)**	**T_**6**_ (*n =* 126)**	**(χ^2^)/ Kruskal-Wallis rank(P value)**
**No. of patients (%)**				
**Administration route**				
Inflorescence^+^	74 (35)	62 (39)	54 (43)	2.58^†^ (0.63)
Oil extract	99 (47)	75 (47)	52 (41)	
Combination*	25 (12)	15 (10)	12 (10)	
Missing N	14	6	8	
**Inflorescence consumption method** ^Ψ^				
Pure MC cigarettes	40 (19)	29 (18)	25 (20)	25.36^†^ (0.91)
MC cigarettes mixed with tobacco	23 (11)	26 (16)	24 (19)	
Other	36 (17)	22 (14)	17 (13)	
**Oil extract consumption method** ^Ψ^				
Sub-lingual	91 (43)	71 (45)	49 (39)	7.25^†^ (0.51)
Swallowing	4 (2)	6 (4)	4 (3)	
**Total dominance of consumed cultivar/s by THC and CBD**				
THC	85 (40)	76 (48)	62 (49)	3.00^†^ (0.56)
CBD	31 (15)	20 (13)	15 (12)	
MIX	48 (23)	30 (19)	25 (20)	
Missing N	48	32	24	
**MC dose (gr)**				
≤ 20	133 (63)	92 (58)	67 (53)	6.01^†^ (<0.05)
≥30	19 (9)	18 (11)	22 (17)	
Missing N	60	48	37	
	**Median (IQR)**	
**Monthly dose (gr)**	20 (20–20)	20 (20–20)	20 (20–20)	8.55^††^ (<0.05)
Missing N	60	48	37	
**THC monthly dose (mg)**	2,000 (1,000–3,000)	2,000 (1,800–3,000)	3,000 (2,000–4,000)	9.12^††^ (<0.01)
Missing N				
**CBD monthly dose (mg)**	1,000 (600–2,000)	800 (600–2,000)	1,200 (600–2,000)	1.22^††^ (0.54)
Missing N	60	48	37	

IQR, Interquartile range; N, Number of patients; +, Inflorescence, MC as flowers consumed by smoking, inhalation, etc.; ^*^ combination refers to patients that consumed both inflorescences and oil extract of MC in the specific time point; T_1_, 1-month Follow-Up; T_3_, 3-month Follow-Up; T_6_, 6-month Follow-Up, endpoint; †, Pearson's Chi-squared test; ††, Kruskal-Wallis rank-sum test; THC, Δ-9-tetrahydrocannabinol; CBD, cannabidiol; Ψ, MC consumption methods do not add up to 100% due to concomitant delivery routes. Percentages are rounded and without decimal points unless <1; gr, grams; mg, milligrams.

### Pain Measures

**Figure 2:**
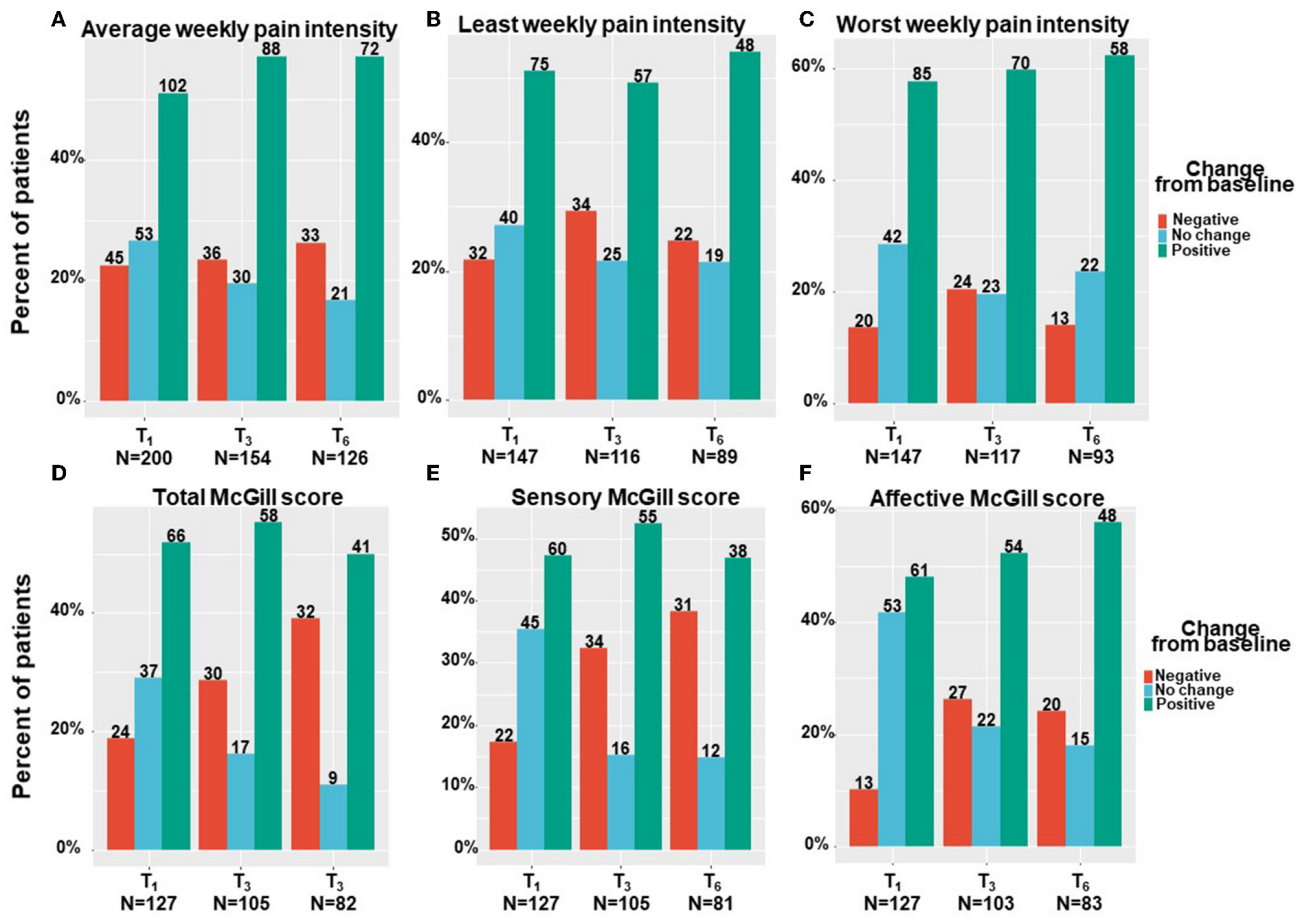
Pain measures change from baseline per time point. T_1_, 1-month Follow-Up; T_3_, 3-month Follow-Up; T_6_, 6-month Follow-Up: Negative indicate patients that reported on higher pain intensity at a follow-up compared to baseline; Positive indicate patients that reported on lower pain intensity at a follow-up compared to baseline; No change indicate patients that reported on the same pain intensity at a follow-up compared to baseline; Numbers of patients are based on patients that reported fully on the measures at baseline and at the corresponding follow-up time point.

**Figure 3:**
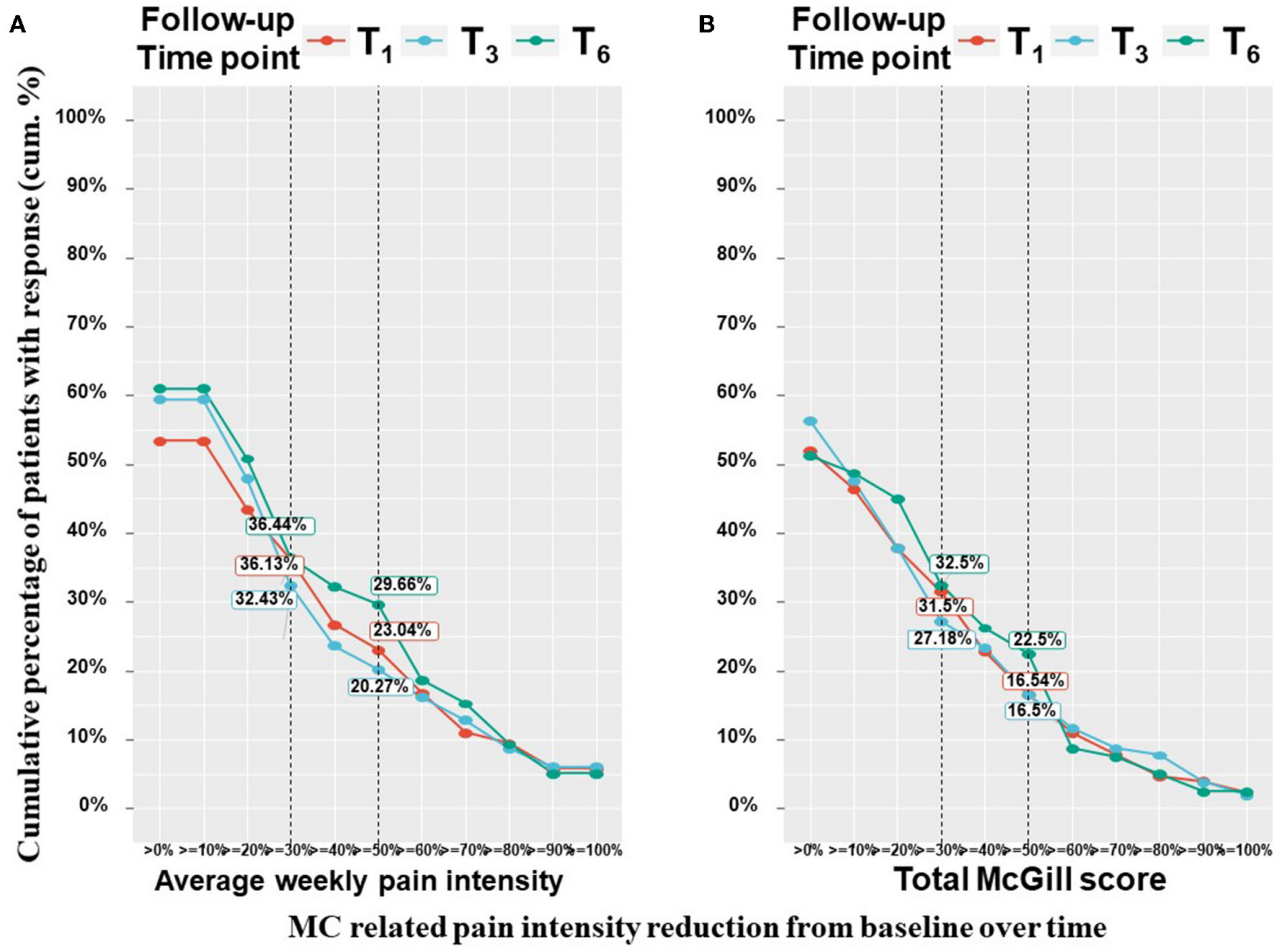
Pain measures cumulative treatment response rates per time point. T_1_, 1-month Follow-Up; T_3_, 3-month Follow-Up; T_6_, 6-month Follow-Up; MC, medical cannabis; The percentages on the Y-axis indicates the cumulative percentage of patients with a response; Every point on the X-axis represent the percentage of MC related pain intensity reduction from baseline, no change values or an increase of pain intensities are not represented in this figure; These figure displays percentages from the entire cohort (*n* = 212 at T_1_, *n* = 158 at T_3_, *n* = 126 at T_6_) but only positive (pain decrease) reports are visible.

Patients have been suffering from pain for 4 (2–4) months at T_0_. All pain measures improved from T_0_ at all the follow–up time points, as revealed by means of linear mixed regression model analyses. Mentionable are the following significant changes between T_0_ and T_6_ for patients that reached the endpoint: average weekly pain intensity reduced by a median of 20% and IQR of 0 to 50% from 7 (3–9) to 5 (3–7) (−0.98; 95%CI = −1.43 to −0.54; *p* < 0.001); least pain intensity declined by a median of 25% and IQR of 0% to 56% from 6 (3–8) to 5 (2–7) (−0.81; 95%CI= −1.40 to −0.21; *p* < 0.01) and worst pain intensity by a median of 20% and IQR of 0 to 43% from 8 (6–10) to 6 (4–8) (−1.78; 95%CI = −2.31 to −1.26; *p* < 0.001). The total SF–MPQ score dropped by a median of 7% and IQR of −17 to 45% from 23 (15–30) to 20 (10–27) (−4.74; 95%CI = −6.80 to −2.68; *p* < 0.001). Within the SF–MPQ, the affective pain components showed a reduction by a median of 20% and IQR of 0 to 56% from 6 (4–8) to 4 (2–7) (−2.08; 95% CI = −2.75 to −1.40; *p* < 0.001) and the sensory pain components by a median of 0% and IQR of −32 to 37% from 17 (10–22) to 16 (8–22) (−2.79; 95%CI = −4.36 to −1.23; *p* < 0.001). The full spectrum of responses for all pain measures is presented in Figure 2; the numbers and percentage of patients reporting positive (i.e., pain decrease), no change or negative (i.e., pain increase) responses at each time point are indicated. While most patients reported some degree of pain intensities decrease, about 20% of patients reported either no change in their pain intensity from baseline or on pain intensity increase. Mentionable, the rates of no change from baseline were higher (35–40%) for the sensory and affective pain intensities. Focusing on the most clinically important measures, the cumulative treatment response rates of average weekly pain intensity and total McGill score are presented in Figure 3. Notably, 36 and 33% of the cohort reported on ≥30% average pain intensity and total SF–MPQ score reduction at T_6_, respectively.

### Analgesics Consumption

Of the patients in the cohort that reported fully on their analgesic medications' consumption at T_0_ and T_6_ (*n* = 126), 40% of those (*n* = 30) who had been using analgesic medications (over-the-counter, non-steroidal anti-inflammatory drugs, opioids, anticonvulsants, and antidepressants) at T_0_ (*n* = 74), were no longer using them. Conversely, ten patients (20%) initiated analgesic medications at T_6_ while not consuming any at T_0_. Specifically, very few patients that survived to T_6_ consumed opioids at T_0_. When translated into morphine equivalent dose, Median (IQR) was 0 (0-0) at both time points.

### Cancer Symptom Burden

**Figure 4:**
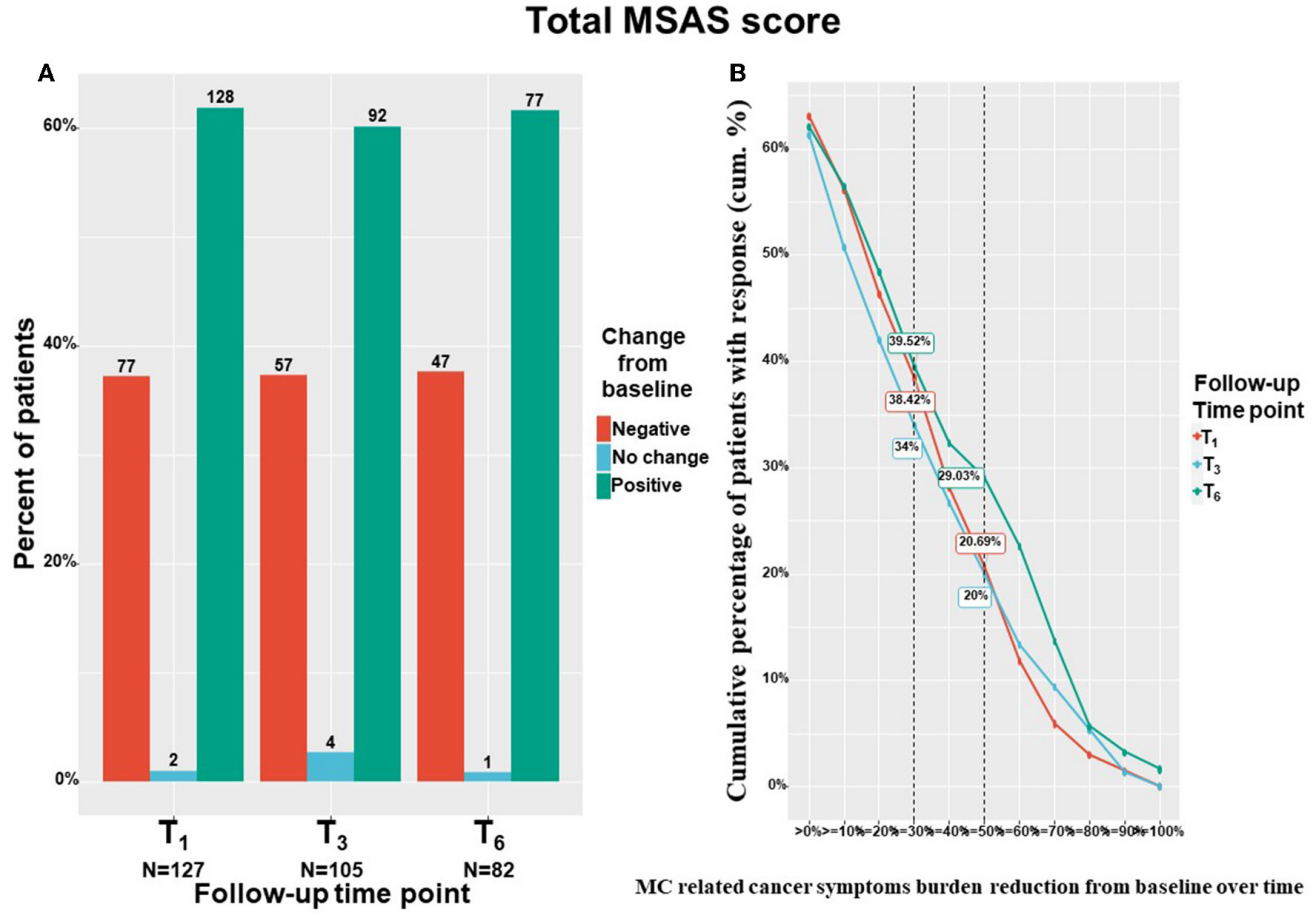
Total cancer symptom burden change from baseline. T_1_, 1-month Follow-Up; T_3_, 3-month Follow-Up; T_6_, 6-month Follow-Up: MSAS, Memorial Symptom Assessment Scale; **(A)** Negative indicate patients that reported on higher pain intensity at a follow-up compared to baseline; Positive indicate patients that reported on lower pain intensity at a follow-up compared to baseline; No change indicate patients that reported on the same pain intensity at a follow-up compared to baseline; MC, medical cannabis; The percentages on the Y-axis indicates the cumulative percentage of patients with response; For **(B)**, every point on the X-axis represent the percentage of MC related pain intensity reduction from baseline, no change values or an increase of pain intensities are not represented in this figure; Numbers of patients are based on patients that reported fully on the measures at baseline and at the corresponding follow-up time point; **(B)** displays percentages from the entire cohort (*n* = 212 at T_1_, *n* = 158 at T_3_, *n* = 126 at T_6_) but only positive (MSAS score improvement) reports are visible.

The study's primary outcome measure, the cancer symptom burden (i.e., MSAS total score), decreased significantly from T_0_ to T_6_ by means of linear mixed regression model analyses. Cancer symptom burden decreased by a median of 18% and IQR of−22% worsening to 57% reduction from 122 (82–157) to 89 (45–138) (−18.98; 95%CI= −26.95 to −11.00; *p* < 0.001). The subscales of the MSAS questionnaire also improved significantly. Specifically, MSAS general distress index decreased by a median of 22% and IQR of −5% worsening to 54% reduction from 52 (34–66) to 34 (18–57) (−10.29; 95%CI= −13.50 to −7.08; *p* < 0.001), physiological index decreased by a median of 18% and IQR of −10% worsening to 60% reduction from 36 (24–52) to 23 (12–44) (−8.24; 95%CI= −11.05 to −5.43; *p* < 0.001) and the psychological index decreased by a median of 18% and IQR of −21% worsening to 51% reduction from 31 (17–41) to 22 (11–36) (−5.81; 95%CI= −7.98 to −3.64; *p* < 0.001). Figure 4A demonstrates the numbers and percentage of patients reporting positive change (e.g., total cancer symptom burden decrease), no change or negative (e.g., total cancer symptom burden increase) change at each time point. Notably, most patients (about 60%) reported a positive effect. Figure 4B demonstrates the cumulative treatment response rates of the total cancer symptom burden. Notably, almost 40% of the cohort reported on ≥30% total cancer symptom burden at T_6_.

### Cancer Comorbid-Related Symptoms

**Figure 5:**
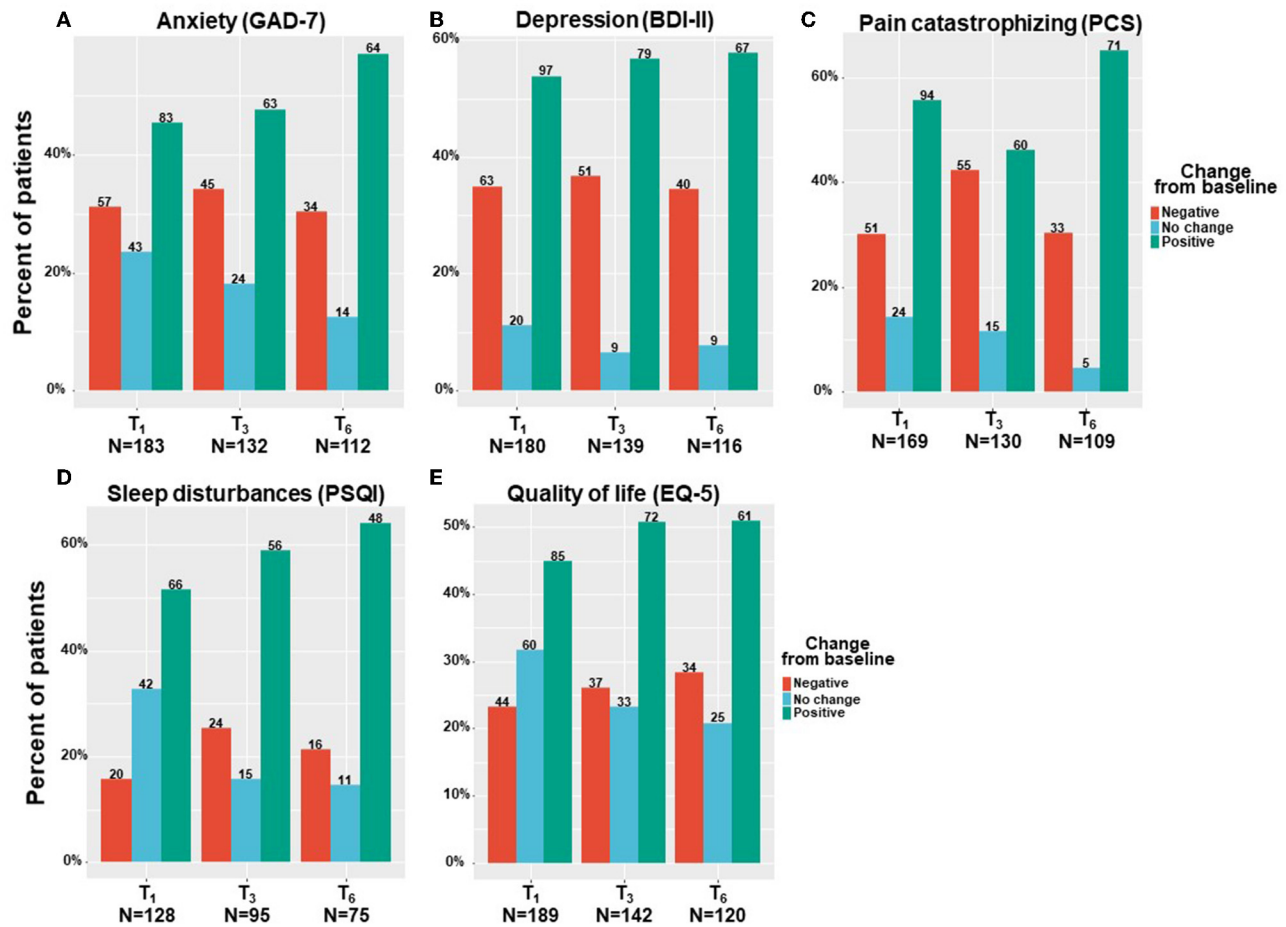
Cancer comorbid symptoms change from baseline. T_1_, 6-month Follow-Up; T_3_, 6-month Follow-Up; T_6_, 6-month Follow-Up: GAD-7, general anxiety disorder; BDI, Beck depression inventory; PCS, pain catastrophizing scale; PSQI, Pittsburgh sleep quality index; EQ-5, Euro-QoL questionnaire; Negative indicates patients that reported on higher comorbid symptoms at a follow-up compared to baseline; Positive indicate patients that reported on lower comorbid symptoms at a follow-up compared to baseline; No change indicate patients that reported on the same comorbid symptoms at a follow-up compared to baseline; Numbers of patients are based on patients that reported fully on the measures at baseline and at the corresponding follow-up time point.

By means of linear mixed regression model analyses, a significant decrease was found in anxiety levels, which decreased by a median of 22% and IQR of −14% worsening to 64% improvement from 9 (3–14) at T_0_ to 6 (2–11) at T_6_ (−2.35; 95%CI= −3.31 to −1.40; *p* < 0.001). Depression severity also decreased by a median of 12% and IQR of −24% worsening to 30% improvement from 19 (11–24) at T_0_ to 15 (10–22) at T_6_ (−1.97; 95%CI= −3.26 to −0.68; *p* < 0.001). Pain catastrophizing scores reduced by a median of 18% and IQR of −10% worsening to 45% improvement, from 30 (19–39) at T_0_ to 24 (12–33) at T_6_ (−5.44; 95%CI= −7.73 to −3.15; *p* < 0.001). Sleep disturbance scores decreased by 16% and IQR of 0% worsening to 43% improvement from 12 (7–15) at T_0_ to 8 (6–12) at T_6_ (−3.07; 95%CI = −3.95 to −2.18; *p* < 0.001). Finally, the quality–of–life score decreased (i.e., improved) significantly from T_0_ to T_6_ by a median of 14% and IQR of −18% worsening to 40% improvement reduction from 4 (3–5) to 4 (2–5) (−0.55; 95%CI= −0.83 to −0.27; *p* < 0.001). According to the IQR range, unlike most comorbid cancer–related symptoms, where the lower limit of the IQR percentage of change response was negative, the IQR sleep disturbance scores showed only positive changes (0–43%). Figure 5 demonstrates the numbers and percentage of patients reporting positive change (e.g., comorbid symptoms decrease), no change, or negative change (e.g., comorbid symptoms increase) at each time point. Notably, most patients (about 60%) reported a positive effect.

### Sexuality Problems

As described in the methods section, sexuality problems were assessed with specific and different validated questionnaires for females and males. After adjusting for the higher proportion of females in the sample, the response rate to the sexuality questionnaires between the genders was similar. Notably, the response rate to these questionnaires was very low (12–17%).

**Figure 6:**
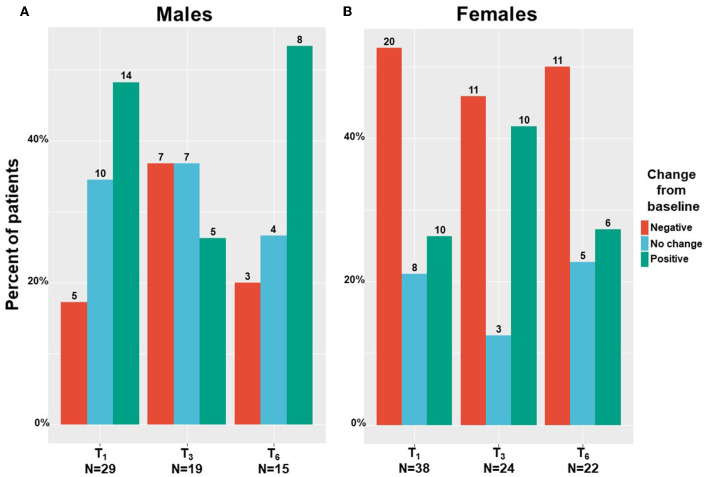
Sexuality problems change during medical cannabis between sexes. T_1_, 1-month Follow-Up; T_3_, 3-month Follow-Up; T_6_, 6-month Follow-Up; numbers on bars represent the number of patients that reported.

We found that males mainly reported on absolute improvement in their sexuality problems following MC treatment (Figure 6A), with scores increased by a median of 6% and IQR of 0% with no change to 29% improvement, from 7 (5–18) at T_0_ to 5 (5–26) at T_6_ (−2.39; 95%CI= −10.65 to 5.86; *p* = 0.52). On the contrary, females reported mainly on absolute worsening in their sexuality problems following MC treatment (Figure 6B), with scores reduced by a median of −2% and IQR of −51% worsening to 8% improvement, from 12 (5–39) at T_0_ to 21 (5–55) at T_6_ (6.99; 95%CI = −2.14 to 16.13; *p* < 0.001). Nonetheless, these changes during treatment were not significant for both sexes.

### Weight Characteristics

Weight and body mass index (BMI) remained unchanged on average, but their ranges had a small change that was statistically significant, from 70 (60–80) kg and 25 (23–29) at T_0_ to 70 (62–80) kg and 25 (22–8) at T_6_ (-0.96; 95%CI= −1.83 to −0.10; *p* < 0.05 and −0.42; 95%CI = −0.73 to −0.10; *p* < 0.01, respectively).

### Medical Cannabis Treatment Safety

**Figure 7:**
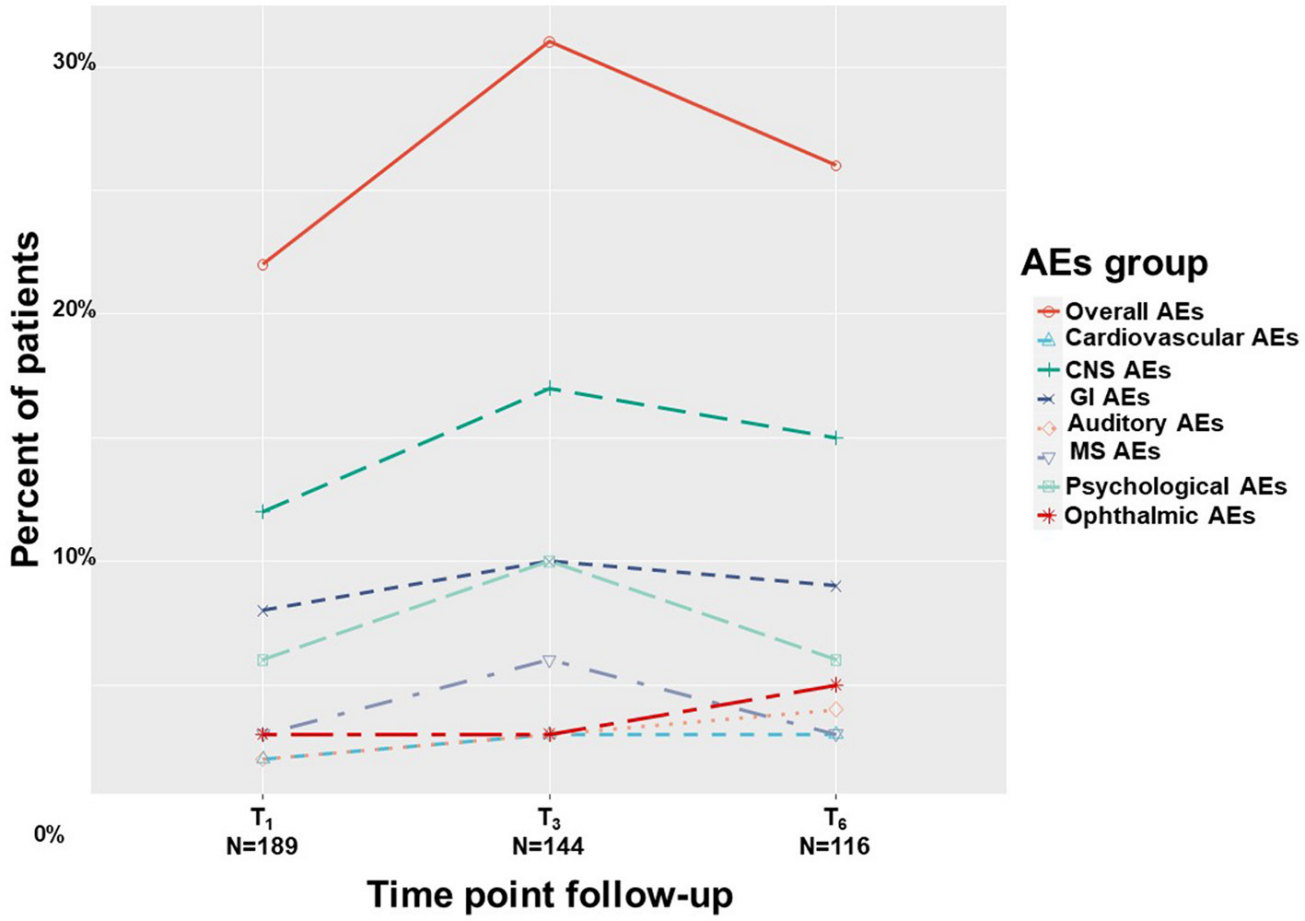
Medical cannabis-related adverse effects. T_1_, 1-month Follow-Up; T_3_, 3-month Follow-Up; T_6_, 6-month Follow-Up; AEs, adverse effects; Overall, at least one AE report; CNS, central nervous system; GI, gastrointestinal; MS, musculoskeletal.

Overall, by means of generalized linear mixed regression model analyses, we found 20%-30% of patients reported on AEs with no significant change across treatment duration, from T_1_ to T_6_ (0.47; 95%CI = −0.29 to 1.24; *p* = 0.22). These AEs were mainly non-serious according to FDA definition (42) and did not cause MC treatment discontinuation. The AEs of the affected systems in descending order of report rates were: central nervous systems (10–17%), gastrointestinal (7–10%), psychological (5–10%), ophthalmic (3–5%), musculoskeletal (3–6%), cardiovascular (1–3%) and auditory (2–4%), all showed no significant change across treatment duration (Figure 7). A total of 36 (11%) patients discontinued MC treatment due to MC-related AEs. The specifics of the AE were unknown for eight of them, the remainder were fatigue (*n* = 5), dizziness (*n* = 4), hallucinations (*n* = 4), bad taste (*n* = 3), drowsiness (*n* = 2), and abdominal pain, anxiety, cough, fainting, heat waves, hypotension, nausea, palpitations, restlessness and shortness of breath (one each). Cessation of treatment was made by patients alone, so no account for the severity of AEs could be done. We were able to verify that patients lost to follow-up did not pass away during the study period.

Notably, 69 (21%) of the patients that initiated MC treatment passed away, while 255 patients survived during the 6-months follow-up period. Several significant differences were found between these groups, presented in Supplementary Table 4.

### Hospitalizations

During an overall period of 9 months, the 3 months preceding T0, and between T0 to either T1, T3, or T6, there were *n* = 80, 25%, *n* = 14, 7%, *n* = 15, 10% and *n* = 25, 20%, hospitalizations due to surgeries, respectively, and *n* = 98, 30%, *n* = 26, 12%, *n* = 21, 13% and *n* = 14, 11%, hospitalizations due to other reasons, respectively. Most surgeries were performed in the 3 months preceding MC treatment initiation and included mostly solid tumor removals. At the follow-ups, most hospitalizations were also for the reason of solid tumor removal. Other reasons for hospitalizations included cancer, treatment diagnosis and oncology treatment complications. Notably, none of the hospitalizations were considered directly related to the MC treatment.

## Discussion

There is a growing interest in studies on the effectiveness of an MC treatment for oncology patients (69). The main finding of the current study is that most cancer comorbid symptoms improved significantly during 6 months of MC treatment. The change in all pain measures was small. However, as placebo effect is mostly achieved within the first 4 to 12 weeks of treatment (70), it is likely that the changes from baseline found after 6 months are not the result of placebo.

Additionally, we found that MC treatment in cancer patients was well tolerated and safe. These findings align with a few previously published prospective studies on cancer patients (17, 71). The added value of the current analysis is the utilization of validated tools, precise MC treatment measurement, multiple follow-up time points and rigorous follow-up on reasons for dropout from the study. These allowed us to assess the real-life effectiveness and safety of MC treatment for palliative oncology patients.

In a previous report by our group (72), we reported the short-term effects of MC treatment. Some of the reported measures did not improve significantly (i.e., weekly least and worst pain intensities, pain catastrophizing, depression, quality of life and anxiety). We presumed that these parameters may require additional treatment duration for effects to be apparent due to their inherent nature. Indeed, most of the examined parameters were revealed as significant after 6 months. Another support for the long-term benefits of MC treatment is corroborated by a recent retrospective analysis that compared cancer patients with MC treatment to without, which demonstrated symptom relief to those with treatment (49). We presented additional, more comprehensive perspectives of the effects of MC on these measures, including comparisons between rates of patients reporting positive (e.g., improvement), no change or negative (e.g., worsening) rates at each time point, as well as cumulative treatment response that was indorsed by Farrar et al. (50) to make data from clinical studies more understandable. We demonstrated MC treatment was helpful for many oncology patients; however, additional studies are needed to better characterize those patients who could benefit from it. Nonetheless, although more than 50% of the patients reported a reduction in pain intensity, and while 40% of the patients discontinued analgesic medications, 2025% reported on pain intensity increase and 20% reported initiation of an analgesic medication following 6 months of MC treatment. These findings suggest either MC treatment was not equally effective in pain intensity reduction in all patients, some patients might have developed tolerance, or the progression of the oncological disease could not be managed by the previously stable analgesic treatment regimen.

Many of the measures that improved are associated with improved quality of life, which may suggest some of the effect of MC treatment on pain intensity was indirect, as previously discussed (51–53). Studies have shown that quality of life in patients that suffer from a severe illness such as cancer plays an important role in treatment adherence and success (54–56). Furthermore, the multifactorial effect on chronic pain comorbidities by measures such as quality of life, disability, sleep, anxiety and others were all previously suggested to indirectly affect the observed reduction in pain intensity (57–59).

As the prevalence of cancer diagnoses in our study was similar to previous studies conducted in Israel (17, 71) and to the general cancer prevalence in the public, it can be assumed that the current study findings could be generalized to oncology patients in Israel.

Medical cannabis has been previously reported as a possible remedy for cachexia and appetite loss (60–63). The current study found that the patients' weight and BMI did not change on average during the 6-month follow-ups, but the range did decrease significantly. As a substantial portion of the cohort was diagnosed with progressed cancer, a weight decline is expected with disease progression.

In the current study, almost half of the sample stopped all analgesic medications following 6 months of MC treatment. One explanation for this could be that MC constituted a substitution analgesic (64, 65). Indeed, previous prospective studies have demonstrated similar findings in chronic non-cancer pain cohorts (57, 58), and in a survey of gynecologic cancer patients, almost half reported that they decreased opioids following MC initiation (66). Another explanation is that the disease of patients that survived the 6 months of MC treatment was milder; they had fewer comorbidities and might also be cancer-free by the endpoint.

In the extended period of 6 months, there were mostly non-serious AEs with no significant change from those at the one-month checkpoint, the most frequent being dizziness and tiredness. This finding aligns with previous studies (57, 71), suggesting these AEs can be attributed to the MC treatment and not to the disease itself. While earlier results of Aviram et al. (57) reported a decrease in MC-related AEs during treatment for chronic non-cancer pain, in the current study of palliative oncology patients, the AEs remained stable during the 6 months of the study. This finding may be attributed to the relatively stable MC treatment dose (20 gr for most patients) in the current study, contrary to a significant dose increase in Aviram's research and a different frequent administration route. Notably, it may be suggested that in the 6 months of MC treatment, patients did not need to increase the dose for MC treatment to be as effective as at the beginning of the treatment. Notwithstanding, in the current study, the monthly dose of THC increased during the 6 months treatment. Similar long-term safety was previously demonstrated in cancer patients (67). It is plausible that THC dose increase in the current study was not associated with elevation of AEs rates because it is unrelated or that patients that started consuming MC products with higher THC concentration are those less susceptible to its effects, as was demonstrated in a study on the sex differences of MC related AEs (68). Nonetheless, we found that THC monthly dose increased significantly in the 6 months of MC treatment from a Median (IQR) of 2,000 mg (1,000–3,000) to 3,000 mg (2,000–4,000). This increase can be explained by the increase in the rate of patients consuming THC-rich MC from 40 to 49%. It is possible patients developed tolerance to THC or that oncologists tread more cautiously at the beginning of the treatment (start low, go slow), starting the dose adjustment from lower THC concentrations and increasing as the treatment progress according to the suggested treatment protocol (73).

## Limitations

This study has a few limitations. First, no control or placebo groups were assigned, and it is hard to isolate a placebo response from a “true” drug effect. Hence, a prudent interpretation of the results is needed. Second, though only validated questionnaires were utilized and patient responses were kept anonymous from their physician, self-report bias may have still occurred. Third, although advanced statistical approaches for missing data imputation were used, they do not entirely protect the results from this shortcoming (47). Fourth, although the current study presents accurate AEs reports, it is possible that an additional unknown number of patients who were lost to follow-up might have discontinued MC use due to AEs. Finally, due to the sizeable dropout rate of 61% at the endpoint, some survival bias can be seen in most measures, because as time progressed patients showed higher response rates for most measures.

## Conclusions

In conclusion, this prospective, comprehensive and large-scale cohort demonstrated an overall mild to modest long-term statistical improvement of all investigated measures including pain, associated symptoms and, importantly, reduction in opioid (and other analgesics) use. It seems that MC treatment is safe for oncology patients, but its efficacy and clinical relevance may be limited. Oncologists should carefully consider the possible benefits of MC treatment to their patients before prescribing it.

## Data Availability Statement

The original contributions presented in the study are included in the article/Supplementary Material, further inquiries can be directed to the corresponding author/s.

## Ethics Statement

The studies involving human participants were reviewed and approved by the institutional Ethics Committee of Haemek Medical Center (#0137-18-EMC) and Galil Medical Center (#0010-19-NHR) approved the study. The patients/participants provided their written informed consent to participate in this study.

## Author Contributions

JA, GL, GB-S, and DM: conceptualization. JA, AO, IC, AL, MAA, GB-S, LA, DG, NM, AK, and AS: data curation. JA and YV: formal analysis and validation. DM: funding acquisition and resources. JA and DM: investigation. JA, GL, GB-S, YV, and DM: methodology. GL and DM: project administration and supervision. JA, YV, SP, and DM: visualization. JA: writing—original draft. JA, GL, YV, AO, SP, IC, AL, MAA, GB-S LA, DG, NM, AK, AS, and DM: writing—review & editing. All authors participated in data collection, discussed the results, and commented on the manuscript. All authors contributed to the article and approved the submitted version.

## Funding

The study was funded by the Evelyn Gruss Lipper Charitable Foundation. This sponsor had no role or influence on the research or this submission.

## Conflict of Interest

The authors declare that the research was conducted in the absence of any commercial or financial relationships that could be construed as a potential conflict of interest.

## Publisher's Note

All claims expressed in this article are solely those of the authors and do not necessarily represent those of their affiliated organizations, or those of the publisher, the editors and the reviewers. Any product that may be evaluated in this article, or claim that may be made by its manufacturer, is not guaranteed or endorsed by the publisher.
