# Supplementary material for: The Effectiveness and Safety of Medical Cannabis for Treating Cancer Related Symptoms in Oncology Patients
Source: Front Pain Res (Lausanne). 2022 May 20;3:861037. doi: 10.3389/fpain.2022.861037 (PMC9163497; doi:10.3389/fpain.2022.861037)
Supplement: Supplementary file 1 [file Data_Sheet_1.docx]

Supplementary Material

**Table S1: STROBE Statement—Checklist of items that should be included in reports** of ***cohort studies***

|  | Item No | Recommendation | Page  No |
| --- | --- | --- | --- |
| **Title and abstract** | 1 | (*a*) Indicate the study’s design with a commonly used term in the title or the abstract | 1 |
|  |  | (*b*) Provide in the abstract an informative and balanced summary of what was done and what was found | 1 |
| Introduction | | |  |
| Background/rationale | 2 | Explain the scientific background and rationale for the investigation being reported | 1-2 |
| Objectives | 3 | State specific objectives, including any prespecified hypotheses | 2 |
| Methods | | |  |
| Study design | 4 | Present key elements of study design early in the paper | 3 |
| Setting | 5 | Describe the setting, locations, and relevant dates, including periods of recruitment, exposure, follow-up, and data collection | 3 |
| Participants | 6 | (*a*) Give the eligibility criteria, and the sources and methods of selection of participants. Describe methods of follow-up | 3 |
|  |  | (*b*) For matched studies, give matching criteria and number of exposed and unexposed | N/A |
| Variables | 7 | Clearly define all outcomes, exposures, predictors, potential confounders, and effect modifiers. Give diagnostic criteria, if applicable | 3 |
| Data sources/ measurement | 8* | For each variable of interest, give sources of data and details of methods of assessment (measurement). Describe comparability of assessment methods if there is more than one group | 4 |
| Bias | 9 | Describe any efforts to address potential sources of bias | 4 |
| Study size | 10 | Explain how the study size was arrived at | 4 |
| Quantitative variables | 11 | Explain how quantitative variables were handled in the analyses. If applicable, describe which groupings were chosen and why | 4 |
| Statistical methods | 12 | (*a*) Describe all statistical methods, including those used to control for confounding | 4 |
|  |  | (*b*) Describe any methods used to examine subgroups and interactions | N/A |
|  |  | (*c*) Explain how missing data were addressed | N/A |
|  |  | (*d*) If applicable, explain how loss to follow-up was addressed | N/A |
|  |  | (*e*) Describe any sensitivity analyses | N/A |
| Results | | |  |
| Participants | 13* | (a) Report numbers of individuals at each stage of study—eg numbers potentially eligible, examined for eligibility, confirmed eligible, included in the study, completing follow-up, and analysed | 4-5 |
|  |  | (b) Give reasons for non-participation at each stage | 5 |
|  |  | (c) Consider use of a flow diagram | 5 |
| Descriptive data | 14* | (a) Give characteristics of study participants (eg demographic, clinical, social) and information on exposures and potential confounders | 4-6 |
|  |  | (b) Indicate number of participants with missing data for each variable of interest | 4-8 |
|  |  | (c) Summarise follow-up time (eg, average and total amount) | 4-8 |
| Outcome data | 15* | Report numbers of outcome events or summary measures over time | 4-8 |
| Main results | 16 | (*a*) Give unadjusted estimates and, if applicable, confounder-adjusted estimates and their precision (eg, 95% confidence interval). Make clear which confounders were adjusted for and why they were included | 4-8 |
|  |  | (*b*) Report category boundaries when continuous variables were categorized | 4-8 |
|  |  | (*c*) If relevant, consider translating estimates of relative risk into absolute risk for a meaningful time period | N/A |
| Other analyses | 17 | Report other analyses done—eg analyses of subgroups and interactions, and sensitivity analyses | N/A |
| Discussion | | |  |
| Key results | 18 | Summarise key results with reference to study objectives | 8 |
| Limitations | 19 | Discuss limitations of the study, taking into account sources of potential bias or imprecision. Discuss both direction and magnitude of any potential bias | 9 |
| Interpretation | 20 | Give a cautious overall interpretation of results considering objectives, limitations, multiplicity of analyses, results from similar studies, and other relevant evidence | 8-9 |
| Generalisability | 21 | Discuss the generalisability (external validity) of the study results | 9 |
| Other information | | |  |
| Funding | 22 | Give the source of funding and the role of the funders for the present study and, if applicable, for the original study on which the present article is based | 10 |

*Give information separately for exposed and unexposed groups.

Table S2: Characteristics of eligible versus non-eligible participants

| **Group** | **Eligible** | | | **Non-eligible** | | **p** | | | **(χ²)^†^/ Kruskal-Wallis rank^††^** |
| --- | --- | --- | --- | --- | --- | --- | --- | --- | --- |
| **Observations** | | | **No of patients** | | | |  | | |
|  | 324 | | | 80 | |  | | |  |
|  | | **Median (IQR)** | | |  | | |  | |
| **Age at BL** | 64 (53-72) | | | 66 (60-75) | | 0.26 | | | 0.21**^††^** |
| Missing | 26 (8) | | | 54 (68) | |  | | |  |
|  | | **No of patients (%)** | | |  | | |  | |
| **Gender at BL** | |  | | |  | | |  | |
| Female | 192 (59) | | | 39 (49) | | 0.12 | | | 2.50**^†^** |
| Male | 132 (41) | | | 41 (51) | |  | | |  |
| Missing | 0 | | | 0 | |  | | |  |
| **Cancer etiologies at BL*** | | | | | | | | | |
| Breast | 89 (27) | | | 7 (9) | | <0.005 | | | 21.0**^†^** |
| Lung | 36 (11) | | | 9 (11) | |  | | |  |
| Colon | 32 (10) | | | 3 (4) | |  | | |  |
| Ovaries | 23 (7) | | | 4 (5) | |  | | |  |
| Other | 138 (43) | | | 54 (68) | |  | | |  |
| Missing | 6 (2) | | | 3 (4) | |  | | |  |
| **Comorbidities** **at BL** | | | | | | | | | |
| Yes | 167 (52) | | | 8 (10) | | 0.80 | | | 0.06**^†^** |
| No | 146 (45) | | | 9 (11) | |  | | |  |
| Missing | 11 (4) | | | 63 (79) | |  | | |  |
| **Overall analgesics consumption at BL** | | | | | | | | | |
| Yes | 194 (60) | | | 4 (5) | | 0.09 | | | 2.90**^†^** |
| No | 119 (37) | | | 8 (10) | |  | | |  |
| Missing | 11 (4) | | | 68 (85) | |  | | |  |

†, Pearson's Chi-squared test; ††, Kruskal-Wallis rank-sum test; *, Pain etiologies refer to patients with chronic pain etiology only from one origin, combinations refer to patients with more than one chronic pain etiology; IQR, Interquartile range; BL, Baseline.

**Table S3: Elaborated cancer characteristics**

| **Parameters** | **T_0_ (n=324)** | **T_1_ (n=212)** | **T_3_ (n=158)** | **T_6_ (n=126)** | **χ² (P value)** |
| --- | --- | --- | --- | --- | --- |
|  | **No. of patients (%)** | | | |  |
| **Solid tumor etiology**† |  |  |  |  |  |
| Breast | 89 (27) | 63 (30) | 54 (34) | 42 (33) | 3.89 (0.27) |
| Colon | 32 (10) | 25 (12) | 15 (10) | 13 (10) | 0.90 (0.82) |
| Lung | 36 (11) | 24 (11) | 12 (8) | 11 (9) | 1.74 (0.63) |
| Ovaries | 23 (7) | 14 (7) | 9 (6) | 9 (7) | 0.14 (0.99) |
| Bone | 25 (8) | 15 (7) | 14 (9) | 12 (10) | 0.80 (0.85) |
| Prostate | 14 (4) | 10 (5) | 9 (6) | 7 (6) | 0.61 (0.89) |
| Pancreas | 14 (4) | 6 (3) | 4 (3) | 1 (<1) | 4.07 (0.25) |
| Stomach | 13 (4) | 8 (4) | 4 (3) | 2 (2) | 2.11 (0.55) |
| Soft tissues | 10 (3) | 9 (4) | 3 (2) | 2 (2) | 2.74 (0.43) |
| Brain | 10 (3) | 4 (2) | 3 (2) | 1 (<1) | 2.47 (0.48) |
| Uterus | 9 (3) | 7 (3) | 6 (4) | 6 (5) | 1.15 (0.76) |
| Lymphoid | 9 (3) | 5 (2) | 4 (3) | 5 (4) | 0.76 (0.86) |
| Bladder | 7 (2) | 2 (<1) | 1 (<1) | 0 | 4.46 (0.22) |
| Skin | 6 (2) | 4 (2) | 4 (3) | 4 (3) | 0.90 (0.82) |
| Head and Neck | 4 (1) | 3 (1) | 1 (<1) | 2 (2) | 0.63 (0.89) |
| Vaginal | 3 (<1) | 2 (<1) | 2 (1) | 1 (<1) | 0.21 (0.98) |
| Kidneys | 3 (<1) | 2 (<1) | 2 (1) | 2 (2) | 0.44 (0.93) |
| Unknown primary adenocarcinoma | 3 (<1) | 1 (<1) | 1 (<1) | 1 (<1) | 0.37 (0.95) |
| Bile tract | 2 (<1) | 2 (<1) | 1 (<1) | 1 (<1) | 0.22 (0.97) |
| Fallopian tubes | 1 (<1) | 0 | 0 | 0 | NA |
| Thyroid | 1 (<1) | 1 (<1) | 1 (<1) | 1 (<1) | 0.52 (0.91) |
| Nonseminomatous germ cell tumors (NSGCTs) | 1 (<1) | 1 (<1) | 0 | 0 | 1.20 (0.75) |
| Testis | 1 (<1) | 0 | 0 | 0 | NA |
| Missing N | 2 | 4 | 4 | 0 |  |
| **Hematological cancer etiology** |  |  |  |  |  |
| Myelodysplastic syndrome | 2 (<1) | 1 (<1) | 1 (<1) | 1 (<1) | 3.11 (0.96) |
| Chronic lymphocytic leukemia | 1 (<1) | 0 | 0 | 0 |  |
| Acute lymphocytic leukemia | 1 (<1) | 0 | 0 | 0 |  |
| Chronic myeloid leukemia | 2 (<1) | 0 | 1 (<1) | 1 (<1) |  |

†, numbers do not add up to 100% due to concomitant treatments; NA, not applicable.

**Table S4: Baseline characteristics comparison between deceased and surviving patients**

| **Parameters** | **Deceased (n=69)** | **Survived (n=255)** | **(χ²)^†^/ Kruskal-Wallis rank^††^(P value)** |
| --- | --- | --- | --- |
|  | **Median (IQR)** | |  |
| Age | 69 (62-76) | 63 (51-70) | 0.25 (<0.005) |
| Quality of life score (EQ-5) | 6 (4-7) | 4 (3-4) | 0.32 (<0.001) |
| Depression score (BDI) | 21 (16-29) | 18 (11-23) | 0.23 (<0.05) |
| ECOG score | 2 (1-2) | 1 (0-1) | 0.35 (<0.001) |
|  | **No. of patients (%)** | |  |
| Any physical activity (yes) | 2 (3) | 68 (27) | 16.00 (<0.001) |
| Metastatic status | 48 (70) | 91 (36) | 23.00 (<0.001) |
| Breast cancer etiology | 9 (13) | 83 (33) | 9.00 (<0.005) |
| Pancreases cancer etiology | 8 (12) | 6 (3) | 9.30 (<0.005) |
| Carboplatin chemotherapy | 14 (20) | 23 (9) | 5.90 (<0.05) |
| Strong opioids consumption | 20 (29) | 44 (17) | 5.40 (<0.05) |

†, Pearson's Chi-squared test; ††, Kruskal-Wallis rank-sum test; BDI, Beck depression inventory; Euro-QoL questionnaire; ECOG, Eastern Cooperative Oncology Group Performance Status; Only statistically significant measures are presented.
